# Subchondral Bone Density Distribution in Canine C6–C7 Vertebral Endplates Affected by DA-CSM: A CT-OAM Study

**DOI:** 10.3390/ani16132098

**Published:** 2026-07-07

**Authors:** Vincenz Kramer, Peter Böttcher

**Affiliations:** 1 Tierklinik Stuttgart Plieningen, 70599 Stuttgart, Germany; 2 Mitteldeutsches Kompetenzzentrum für Kleintiere, 04316 Leipzig, Germany; peter.boettcher@mkk-leipzig.de

**Keywords:** CT-OAM, canine, cervical spine, bone density, cervical spondylomyelopathy, Wobbler syndrome, subchondral bone, endplate, disease-associated remodelling, subsidence

## Abstract

Disc-associated cervical spondylomyelopathy (DA-CSM) is a clinically relevant disorder of the canine cervical spine frequently requiring surgical stabilization, with implant subsidence remaining a common complication. This study aimed to evaluate subchondral bone mineral density (sBMD) distribution in the C6–C7 vertebral motion unit of DA-CSM-affected dogs and to compare these findings with a previously established cohort of clinically unaffected dogs. Computed tomography osteoabsorptiometry (CT-OAM) was applied to eight affected specimens, and sBMD was analyzed across predefined annulus fibrosus (AF) and nucleus pulposus (NP) regions, including detailed topographic subdivisions. Statistical comparisons were performed using paired and independent t-tests with correction for multiple testing, while the topographic pattern of bone density minima and maxima was compared using Chi-squared testings. DA-CSM-affected vertebral endplates demonstrated a consistent and significant reduction in overall sBMD compared to controls (*p* < 0.0001), affecting both AF and NP regions as well as all topographic subdivisions. Despite this global decrease, the characteristic spatial distribution pattern was not significant different between affected and unaffected vertebrae, with higher sBMD values in peripheral AF regions and lower values in central and centro-dorsal NP regions. These findings indicate that DA-CSM is associated with a generalized reduction in subchondral bone density without alteration of the intrinsic load-adapted distribution pattern. The persistence of structurally weak central regions, combined with reduced overall bone quality, may contribute to the risk of implant subsidence. Consideration of both global and regional sBMD characteristics may therefore be relevant for optimizing implant design and surgical strategies in canine cervical spinal distraction and stabilization.

## Simple Summary

Surgical treatment of disc-associated cervical spondylomyelopathy in dogs frequently involves placement of intervertebral implants to decompress and stabilize the cervical spine. A major complication of these procedures is implant subsidence, in which the implant gradually sinks into the adjacent vertebral bone. This complication may be influenced by the subchondral bone mineral density of the vertebral endplates that support the implant. The present study investigated the distribution of subchondral bone mineral density within the C6–C7 vertebral endplates in dogs affected by spondylomyelopathy and compared the findings with those from clinically unaffected dogs. Using computed tomography osteoabsorptiometry, we identified a marked and consistent reduction in overall subchondral bone density in affected dogs across all evaluated endplate regions, indicating a generalized decrease in structural bone quality in diseased vertebral endplates. Despite this global reduction, the topographic bone density distribution remained unchanged. Peripheral annulus fibrosus regions consistently exhibited the highest bone density values, whereas the central and centro-dorsal nucleus pulposus regions showed the lowest density values in both affected and unaffected dogs. These low-density central regions correspond to mechanically vulnerable areas that are commonly associated with implant subsidence. The findings suggest that dogs affected by cervical spondylomyelopathy may have an increased biomechanical risk of implant subsidence. Nevertheless, although overall subchondral bone quality is reduced, the regional pattern of density distribution within the vertebral endplates remains preserved and may therefore guide future spinal implant design.

## 1. Introduction

DA-CSM represents a clinically significant and biomechanically complex disorder of the canine cervical spine, frequently necessitating surgical intervention to achieve decompression and stabilization [1,2]. Despite advances in surgical techniques, including intervertebral cage placement and ventral fixation, complications such as cage subsidence remain highly prevalent and continue to compromise clinical outcomes [3,4,5,6,7,8,9].

In a previous study, CT-OAM was used to characterize the sBMD distribution within the caudal endplate of C6 and the cranial endplate of C7 in clinically and radiologically unaffected dogs. That investigation revealed a distinctly heterogeneous density pattern, with reduced sBMD in the central and centro-dorsal regions corresponding to the NP contact area, and higher density values located predominantly in the peripheral AF regions. Importantly, these low-density regions coincided with the commonly reported sites of cage subsidence, suggesting a direct biomechanical relevance of sBMD distribution for surgical planning and implant design [10].

However, the applicability of these findings to clinical cases of DA-CSM remains uncertain. Pathological alterations associated with the disease, including endplate sclerosis, disc degeneration, and chronic abnormal loading, may substantially modify the subchondral bone architecture. Such changes could alter both the absolute sBMD values and their spatial distribution, thereby influencing the mechanical competence of the vertebral endplates and the risk profile for implant subsidence.

In human spinal research, disease-associated remodelling of subchondral bone has been shown to significantly affect local bone density patterns and mechanical properties [11]. Comparable processes are likely to occur in dogs affected by DA-CSM, yet no data currently exist describing sBMD distribution in diseased canine cervical vertebral endplates.

Therefore, the primary objective of the present study was to characterize the subchondral bone mineral density distribution in the C6–C7 vertebral motion unit of dogs diagnosed with DA-CSM using CT-OAM. A secondary objective was to directly compare these findings with previously established reference data from clinically and radiologically unaffected dogs. By providing disease-specific information on vertebral endplate bone density, this study also aims to contribute to the refinement of surgical strategies, optimization of implant positioning, and development of next-generation intervertebral cages tailored to the pathological biomechanics of the canine vertebral spine. We hypothesized that vertebral endplates affected by DA-CSM would exhibit altered sBMD distribution patterns compared with clinically unaffected dogs, particularly in regions subjected to chronic pathological loading, potentially resulting in either localized increases due to sclerosis or decreases due to structural weakening.

## 2. Materials and Methods

### 2.1. Case Selection

Cases were retrospectively selected from two institutions: the Tierklinik Nürnberg Hafen and the Freie Universität Berlin (Clinic for Small and Companion Animals). Inclusion criteria required a confirmed diagnosis of DA-CSM affecting the C6–C7 vertebral motion unit, established by computed tomography (CT). In addition, all dogs had to present with chronic clinical signs consistent with DA-CSM and a neurological status of at least grade 1 according to the modified Frankel score.

Exclusion criteria comprised the presence of concurrent vertebral neoplasia, fractures, congenital malformations, discospondylitis, or any other condition that could independently influence vertebral bone density. Furthermore, cases with severe motion artifacts or incomplete imaging data were excluded.

For all included cases, signalment data (age, breed, sex, and body weight) were recorded where available.

### 2.2. CT Data Acquisition and Processing

Computed tomography datasets were acquired as part of the clinical diagnostic work-up at the respective institutions. Although minor variations in scanning protocols existed between the two centers, all scans were performed using multi-slice CT systems with a slice thickness of ≤2 mm, allowing high-resolution assessment of the vertebral endplates.

Image processing and analysis were performed in analogy to the previously described workflow in clinically unaffected dogs [10]. CT datasets were exported in DICOM format and imported into MeVisLab (Version 3.5.0; MeVis Medical Solutions AG, Bremen, Germany) for isolation of the region of interest. The caudal endplate of C6 and the cranial endplate of C7 were manually segmented and extracted.

For subsequent analysis, all endplates were spatially matched to the reference endplate models established in the previous study to ensure topographic correspondence. Non-rigid image registration was performed using 3D Slicer (Version 5.2.2) [12] in combination with the Elastix module (Version357aaa8) [13,14]. sBMD distribution was assessed using CT-OAM, following the identical protocol described in the reference study. CT-OAM analysis was conducted in ParaView (Version 5.11.0, Kitware Inc., Clifton Park, NY, USA), where maximum intensity projection (MIP) was applied to extract CT attenuation values from the subchondral bone layer. These values, expressed in Hounsfield Units (HU), were projected onto the corresponding reference endplate surfaces, ensuring point-by-point comparability across all specimens.

To enable direct comparison with the previously analyzed healthy cohort, the same anatomical partitioning scheme was applied. Based on prior MRI-based segmentation, each endplate was divided into regions corresponding to the AF and NP, which were further subdivided into predefined topographic regions [10].

### 2.3. Statistical Analysis

Normality of the data was assessed using the Shapiro–Wilk test due to the small sample size. CT-OAM values are presented as mean ± standard deviation (SD), as well as minimum and maximum values where appropriate.

For comparisons between affected and unaffected groups, independent-samples t-tests were applied for normally distributed data with equal variances.

Topographic comparisons within vertebral endplates, including comparisons between predefined subdivisions, were performed using paired-samples t-tests for normally distributed data.

To account for multiple comparisons across predefined topographic subdivisions, a Bonferroni correction was applied. Accordingly, the level of statistical significance was set at *p* ≤ 0.05 for primary comparisons and adjusted to *p* ≤ 0.01 for analyses involving multiple regional comparisons.

For additional analysis of topographic distribution patterns, the predefined functional sum-group regions were categorized into three ordinal density classes based on tertile thresholds derived separately for each vertebral level and study group. Regions with mean sBMD values below the 33rd percentile were classified as low density (category 0), regions above the 66th percentile as high density (category 2), and values between both thresholds as intermediate density (category 1). Frequency distributions of these categorical assignments were subsequently compared between DA-CSM-affected and clinically unaffected VMUs using chi-squared tests. Statistical significance was defined as *p* ≤ 0.05.

All statistical analyses were performed using the MedCalc Statistical Software (Version 23.4.9; MedCalc Software Ltd., Ostend, Belgium).

## 3. Results

### 3.1. Findings in DA-CSM Affected VMUs

A total of eight canine cervical vertebral motion units (VMUs) from client-owned dogs diagnosed with disc-associated cervical spondylomyelopathy (DA-CSM, Wobbler syndrome) were included in the study. The sample comprised seven male dogs (all castrated) and one spayed female. The mean age was 7.3 years (range: 6–12 years), and the mean body weight was 35.9 kg (range: 21.9–57.0 kg). The study population consisted of two Doberman Pinschers, one Bernese Mountain Dog, one Labrador Retriever, one Rottweiler, one Belgian Malinois, one Small Münsterländer, and one Dalmatian.

**Figure 1:**
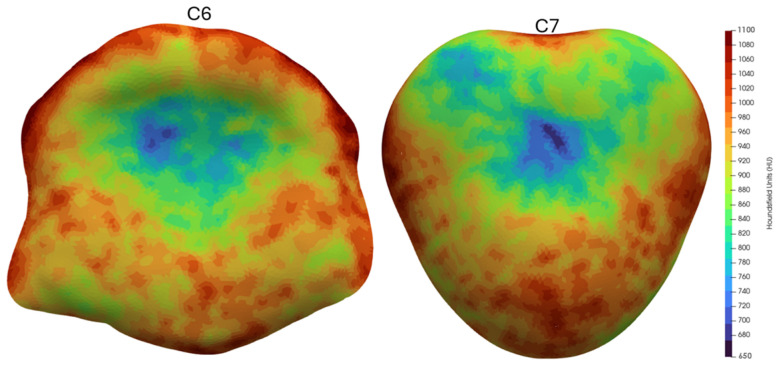
Color mapping of mean sBMD distribution of the caudal endplate of C6 (**left**) and the cranial endplate of C7 (**right**) of DA-CSM affected dogs (*n* = 8).

The sBMD of the caudal endplate of C6 and the cranial endplate of C7 exhibited a heterogeneous distribution pattern, with lower values centrally and higher values in the peripheral regions (Figure 1). The mean sBMD was 938.5 HU (SD: 117.5) for C6 and 923.0 HU (SD: 116.0) for C7.

**Figure 2:**
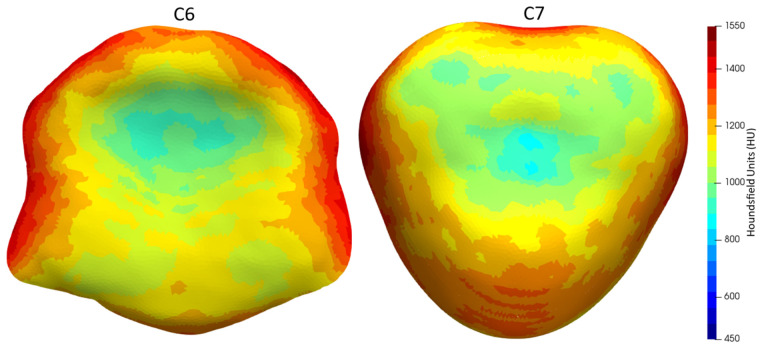
Color mapping of mean sBMD distribution of the caudal endplate of C6 (**left**) and the cranial endplate of C7 (**right**) of the control group (*n* = 15) [10].

For both vertebral endplates, the annulus fibrosus (AF) regions demonstrated higher sBMD values than the nucleus pulposus (NP) regions. In C6, mean sBMD was 977.6 HU (SD: 105.9) in the AF and 900.5 HU (SD: 139.4) in the NP. In C7, corresponding values were 952.6 HU (SD: 118.7) for the AF and 902.4 HU (SD: 122.0) for the NP.

**Figure 3:**
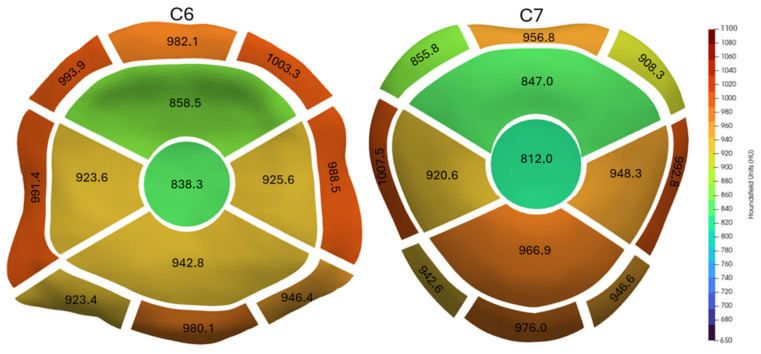
The mean sBMD for the topographic subdivisions of the caudal endplate of C6 (**left**) and the cranial endplate of C7 (**right**) of DA-CSM affected dogs (*n* = 8).

Within the predefined topographic subdivisions (Figure 3), regional variations in sBMD were observed in both vertebral endplates (see Table 1 and Table 2). In C6, the highest values were recorded in the right dorsolateral AF region (1003.3 HU, SD: 115.3), followed by the left dorsolateral (993.9 HU, SD: 137.9) and left lateral AF regions (991.4 HU, SD: 110.7). The lowest values were observed in the central NP region (838.3 HU, SD: 131.9) and the dorsal NP region (858.5 HU, SD: 145.5). No significant differences were detected between the left and right lateral AF regions (left: 991.4 HU, SD: 110.7; right: 988.5 HU, SD: 121.7; *p* = 0.8666) or between the left and right dorsolateral AF regions (left: 993.9 HU, SD: 137.9; right: 1003.3 HU, SD: 115.3; *p* = 0.5567).

**Figure 4:**
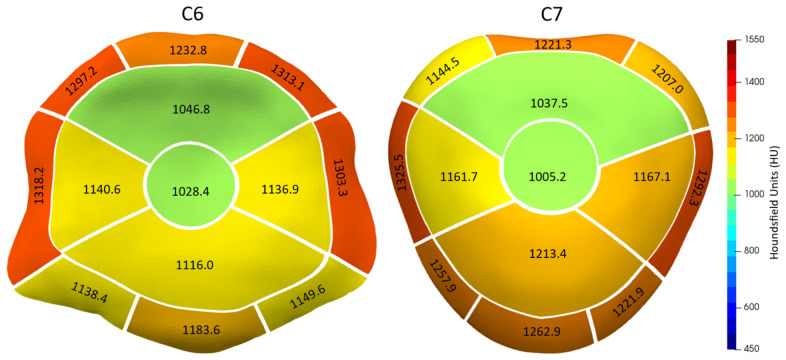
The mean sBMD for the topographic subdivisions of the caudal endplate of C6 (**left**) and the cranial endplate of C7 (**right**) of the control group (*n* = 15) [10].

In C7, the highest sBMD values were found in the right lateral AF (1007.5 HU, SD: 148.9) and left lateral AF regions (992.8 HU, SD: 155.0), whereas the lowest values were observed in the central NP (812.0 HU, SD: 110.3) and dorsal NP regions (847.0 HU, SD: 137.4). A significant difference was observed between the left and right dorsolateral AF regions (left: 908.3 HU, SD: 123.2; right: 855.8 HU, SD: 89.9; *p* = 0.0207), whereas no significant difference was found between the left and right lateral AF regions (left: 992.8 HU, SD: 155.0; right: 1007.5 HU, SD: 148.9; *p* = 0.2866).

**Figure 5:**
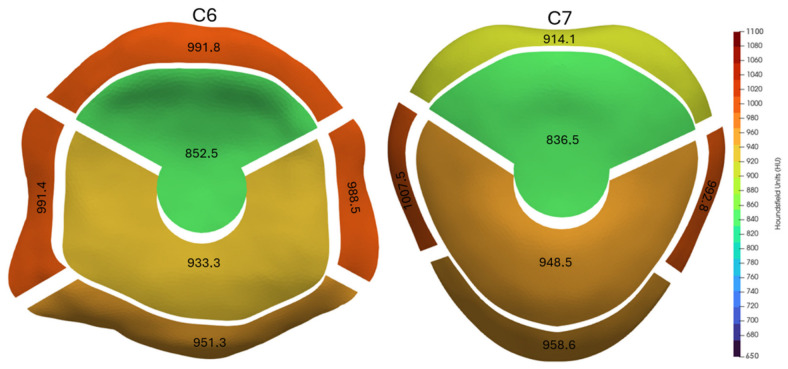
Mean sBMD for the aggregate groups of the subdivisions from Figure 2 with the caudal endplate of C6 (**left**) and the cranial endplate of C7 (**right**) of DA-CSM affected dogs (*n* = 8).

When grouped into larger functional units (Figure 5), the dorsal AF region of C6 exhibited a mean sBMD of 991.8 HU (SD: 127.7), compared to 914.1 HU (SD: 109.7) in C7. The ventral AF region showed mean values of 951.3 HU (SD: 115.8) for C6 and 958.6 HU (SD: 126.9) for C7. Within the NP, the centro-dorsal regions demonstrated mean sBMD values of 852.5 HU (SD: 140.9) for C6 and 836.5 HU (SD: 128.0) for C7, whereas the ventro-lateral regions showed higher values of 933.3 HU (SD: 140.3) and 948.5 HU (SD: 123.7), respectively. 

**Figure 6:**
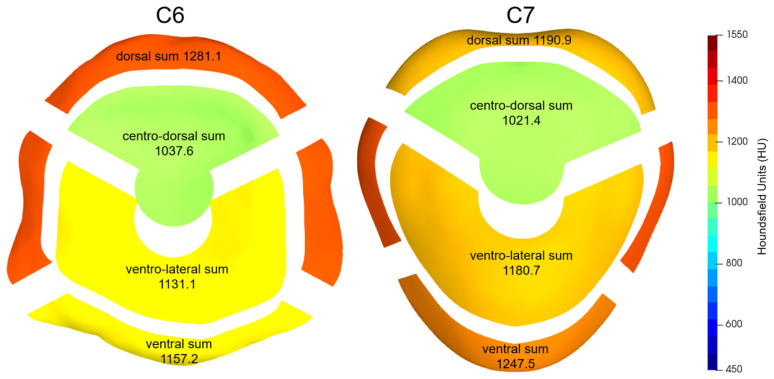
Mean sBMD for the aggregate groups of the subdivisions from Figure 2 with the caudal endplate of C6 (**left**) and the cranial endplate of C7 (**right**) of the control group (*n* = 15) [10].

There was no significant difference in mean sBMD between the C6 (938.5 HU, SD: 117.5) and C7 (923.0 HU, SD: 116.0) endplates (*p* = 0.2292). Likewise, no significant differences were observed between the AF regions of C6 (977.6 HU, SD: 105.9) and C7 (952.6 HU, SD: 118.7) (*p* = 0.1370), or between the NP regions of C6 (900.5 HU, SD: 139.4) and C7 (902.4 HU, SD: 122.0) (*p* = 0.8914).

Within C6, the AF region (977.6 HU, SD: 105.9) showed significantly higher sBMD values than the NP (900.5 HU, SD: 139.4) region (*p* = 0.0274), whereas no significant difference was observed between the dorsal (991.8 HU, SD: 127.7) and ventral (951.3 HU, SD: 115.8) AF regions (*p* = 0.3871). The ventro-lateral NP regions demonstrated significantly higher sBMD values (933.3 HU, SD: 140.3) than the centro-dorsal regions (852.5 HU, SD: 140.9; *p* = 0.0003).

In C7, no significant differences were detected between AF (952.6 HU, SD: 118.7) and NP (902.4 HU, SD: 122.0) regions (*p* = 0.0741) or between dorsal (914.1 HU, SD: 109.7) and ventral (958.6 HU, SD: 126.9) AF regions (*p* = 0.1037). However, the ventro-lateral NP regions showed significantly higher sBMD values (948.5 HU, SD: 123.7) than the centro-dorsal regions (836.5 HU, SD: 128.0; *p* = 0.0011).

### 3.2. Comparison Between DA-CSM-Affected and Clinically Unaffected VMUs

The DA-CSM-affected cohort was compared with a previously published control cohort (Figure 2, Figure 4 and Figure 6) consisting of 15 C6–C7 vertebral motion units obtained from adult canine cadavers weighing between 20 and 45 kg. All control specimens originated from client-owned dogs euthanized for reasons unrelated to the study and had no known conditions affecting bone density. Only VMUs free of CT-detectable or gross anatomical pathology were included.

Compared to the clinically unaffected cohort, DA-CSM-affected vertebral endplates exhibited significantly lower sBMD values across all evaluated regions.

The mean sBMD of the caudal endplate of C6 was significantly reduced in the affected group (938.6 HU) compared to controls (1169.9 HU; *p* < 0.0001). Similarly, the cranial endplate of C7 showed significantly lower sBMD values in affected dogs (923.1 HU) compared to unaffected dogs (1172.3 HU; *p* < 0.0001).

At the regional level, both AF and NP areas demonstrated significantly reduced sBMD values in the DA-CSM group. In C6, the AF region (977.5 HU) and NP region (900.4 HU) were significantly lower compared to controls (AF: 1248.5 HU; NP: 1092.6 HU; *p* ≤ 0.0004). Corresponding findings were observed in C7, with significantly reduced sBMD in both AF (952.6 HU vs. 1245.5 HU; *p* < 0.0001) and NP regions (902.5 HU vs. 1121.0 HU; *p* = 0.0001).

Across all predefined topographic subdivisions, sBMD values were consistently lower in DA-CSM-affected endplates. In the AF regions of C6, all subdivisions—including dorsal, lateral, dorsolateral, and ventrolateral areas—showed significantly reduced sBMD compared to controls (*p* ≤ 0.0008). Similarly, all NP subdivisions of C6 demonstrated significantly lower values (*p* ≤ 0.0013). Equivalent patterns were observed in C7, where all AF and NP subdivisions exhibited significantly reduced sBMD values in the affected group compared to the clinically unaffected cohort (*p* ≤ 0.0008 for NP regions and *p* < 0.0001 for AF regions).

When grouped into functional units, both dorsal and ventral AF regions, as well as centro-dorsal and ventro-lateral NP regions, showed significantly reduced sBMD in affected dogs for both vertebral levels (all *p* ≤ 0.0009).

### 3.3. Topographic Classification of sBMD Distribution

For descriptive analysis of relative density patterns, the predefined functional sum-group regions were categorized into low-, intermediate-, and high-density classes using a tertile-based approach. Thresholds were defined by the 33rd and 66th percentiles calculated separately for each vertebral level and study group, allowing comparison of relative topographic distribution patterns independently of the observed global differences in absolute sBMD values between groups.

In clinically unaffected VMUs, regions of highest sBMD were consistently located within the peripheral annulus fibrosus (AF), particularly the lateral and dorsolateral AF regions of both C6 and C7. In contrast, the lowest sBMD values were consistently identified within the central and centro-dorsal nucleus pulposus (NP) regions. Intermediate values were predominantly observed in the ventral AF and ventro-lateral NP regions. The corresponding tertile thresholds were 1093 HU and 1249 HU for C6, and 1121 HU and 1246 HU for C7.

A comparable topographic distribution pattern was observed in DA-CSM-affected VMUs despite the globally reduced absolute sBMD values. Regions of maximum density remained predominantly located within the lateral and dorsolateral AF regions, whereas minimum density values continued to occur within the central and centro-dorsal NP regions. Intermediate-density classifications were again primarily observed in the ventral AF and ventro-lateral NP regions. The corresponding tertile thresholds in the DA-CSM group were 902 HU and 981 HU for C6, and 903 HU and 966 HU for C7.

Comparison of categorical frequency distributions between affected and clinically unaffected VMUs using chi-squared analysis revealed no significant differences for the dorsal AF, ventral AF, ventro-lateral NP, or lateral AF regions at either vertebral level (all *p* > 0.05). A significant difference was identified only for the centro-dorsal NP region of C6 (*p* = 0.0394), in which DA-CSM-affected VMUs more frequently exhibited classifications within the lowest density category (see Table 3). A similar trend was observed in the centro-dorsal NP region of C7, although this did not reach statistical significance (*p* = 0.0862). Overall, these findings indicate a largely similar pattern of the topographic sBMD distribution in DA-CSM C6-C7 VMUs, despite a marked global reduction in absolute bone density.

## 4. Discussion

The present study investigated the subchondral bone mineral density (sBMD) distribution within the caudal endplate of C6 and the cranial endplate of C7 in dogs affected by disc-associated cervical spondylomyelopathy (DA-CSM) using CT-OAM and compared these findings with a previously established cohort of clinically unaffected dogs. The results demonstrate a consistent and significant reduction in overall sBMD in DA-CSM-affected vertebral endplates, while the characteristic topographic distribution pattern was preserved, with higher sBMD values in peripheral annulus fibrosus (AF) regions and lower values in central and centro-dorsal nucleus pulposus (NP) regions.

The observed global reduction in sBMD across all evaluated regions represents a central finding of the present study. Importantly, this decrease was not restricted to isolated NP regions but affected virtually all functional subdivisions of both vertebral endplates, including the mechanically more robust peripheral AF regions. Independent group comparisons demonstrated significantly lower sBMD values in DA-CSM-affected dogs across all evaluated AF and NP subdivisions of C6 and C7, indicating that the observed changes reflect a generalized alteration of subchondral bone properties rather than focal degenerative remodelling alone.

Despite this pronounced reduction in absolute density values, the relative spatial organization of sBMD remained remarkably consistent between affected and clinically unaffected vertebral motion units. In both cohorts, the highest density values were localized predominantly within the lateral and dorsolateral AF regions, whereas the lowest density values occurred mainly within the central and centro-dorsal NP regions. This preservation of the overall topographic pattern was further supported by the tertile-based classification analysis. Chi-squared comparison of categorized sum-group regions revealed no significant differences between affected and unaffected VMUs for all but one subdivision, including the dorsal AF, ventral AF, lateral AF, and ventro-lateral NP regions of both vertebral levels. Only the centro-dorsal NP region of C6 demonstrated a significant shift toward the lowest density category in affected dogs, while a comparable trend in C7 did not reach statistical significance. Collectively, these findings indicate that DA-CSM is associated predominantly with a uniform downward shift in sBMD rather than with a fundamental redistribution of load-adapted regions.

This distinction is biomechanically relevant. If disease progression had induced substantial redistribution of mechanical loading, one would expect corresponding relocation of density maxima and minima within the vertebral endplates. Instead, the persistence of density maxima in the peripheral AF and density minima in the central NP suggests that the fundamental loading architecture of the C6–C7 motion segment remains largely conserved despite degenerative disease. The significant reduction in absolute density values therefore appears to reflect altered bone quality and remodelling activity superimposed on an otherwise preserved biomechanical loading pattern.

A comparable phenomenon has been described in canine medial coronoid disease and in human synovial joints, where globally reduced subchondral bone density has been associated with persistent remodelling under altered mechanical conditions [15,16]. In these contexts, repetitive microdamage and chronic overload are thought to induce sustained bone turnover, ultimately resulting in reduced mineralization despite ongoing or even increased mechanical demand. By analogy, a similar mechanism may be operative in DA-CSM. Chronic intervertebral disc degeneration, altered cervical kinematics, and abnormal load transmission may promote continuous remodelling activity within the vertebral endplates, thereby reducing overall mineral density while largely preserving the established topographic loading pattern.

From a biomechanical perspective, the persistence of low-density regions within the central and centro-dorsal NP zones is of particular clinical importance. In both affected and unaffected dogs, these regions represented the structurally weakest areas of the endplate and consistently exhibited the lowest sBMD values. Notably, the categorical analysis demonstrated that DA-CSM-affected dogs showed an even greater tendency toward classification within the lowest density category in the centro-dorsal NP region, particularly in C6. These findings are highly relevant because these central NP regions correspond closely to the commonly reported locations of intervertebral cage subsidence following surgical stabilization [4,7,17,18]. The present data therefore suggest that disease-associated remodelling does not compensate for intrinsic endplate weak points but instead occurs within an already mechanically vulnerable topographic framework.

Clinically, these findings may have direct implications for surgical planning and implant design. The combination of globally reduced sBMD and persistent central low-density regions may substantially increase susceptibility to implant subsidence in DA-CSM-affected dogs. Even when implants are positioned toward relatively denser peripheral AF regions, the overall reduction in endplate bone quality may compromise load-bearing capacity. Consequently, implant concepts that merely target local density maxima may be insufficient if they fail to account for the generalized reduction in structural competence.

The preservation of the topographic distribution pattern may, however, provide an important opportunity for optimization of implant geometry. Because the spatial arrangement of mechanically stronger and weaker regions remains largely unchanged, implant designs could potentially be adapted specifically to the preserved density architecture of the canine cervical endplate. Intervertebral cages with enlarged peripheral load-bearing surfaces, reduced central stress concentration, or patient-specific contact geometries may therefore improve load transfer and decrease the risk of subsidence. The present findings thus support the concept that endplate-specific density mapping may represent a valuable basis for future implant development in canine cervical spinal surgery.

Several limitations of the present study should be considered when interpreting the findings. First, the relatively small sample size reflects the limited availability of well-characterized clinical cases with sufficient CT image quality for CT-OAM analysis and may restrict statistical power, particularly for subgroup comparisons. Second, although all datasets fulfilled predefined quality criteria, minor differences in CT acquisition parameters between institutions cannot be entirely excluded and may have influenced absolute Hounsfield Unit values. Nevertheless, the use of a standardized registration and processing workflow likely minimized these effects and supports the comparability of relative regional distribution patterns.

Furthermore, the retrospective nature of the study precluded full control over potentially confounding variables such as breed-specific morphology, body weight, and disease severity. These factors may contribute to inter-individual variation in sBMD and should be addressed in future prospective investigations. Importantly, the cross-sectional design does not permit conclusions regarding the temporal progression or causality of the observed alterations. It therefore remains unclear whether reduced sBMD represents a primary predisposing factor, an adaptive remodelling response, or a consequence of chronic degeneration. Longitudinal studies will be necessary to clarify these relationships.

Finally, CT-OAM provides an indirect assessment of subchondral bone mineralization based on CT attenuation values and does not capture microarchitectural characteristics of bone tissue. Complementary histological, biomechanical, or micro-CT investigations would therefore be valuable to further characterize the structural basis of the observed density alterations.

Despite these limitations, the present study provides novel insights into disease-associated alterations of subchondral bone in the canine cervical spine. The findings demonstrate that DA-CSM is characterized by a marked global reduction in vertebral endplate sBMD while maintaining the fundamental topographic organization of density distribution. Future biomechanical studies integrating implant testing and failure analysis may further elucidate how these alterations influence endplate competence and subsidence risk in surgically treated dogs.

## 5. Conclusions

In conclusion, the present study demonstrates a consistent reduction in overall sBMD in the vertebral endplates of dogs affected by DA-CSM compared to clinically unaffected controls. Despite this global decrease in bone density, the characteristic topographic distribution pattern remained largely preserved, with density maxima consistently located within the peripheral annulus fibrosus regions and density minima within the central and centro-dorsal nucleus pulposus regions.

These findings indicate that DA-CSM is associated primarily with a generalized reduction in endplate bone quality rather than a fundamental redistribution of biomechanical loading zones. Consequently, regions that already represent structurally vulnerable areas in clinically unaffected dogs remain the mechanically weakest regions in diseased vertebral motion units.

From a clinical and biomechanical perspective, this observation is highly relevant for surgical stabilization procedures involving intervertebral cages. The persistence of low-density central NP regions, combined with an overall reduction in sBMD, may contribute substantially to the high incidence of implant subsidence reported in DA-CSM surgery. The preserved spatial organization of density patterns suggests that future implant designs may benefit from specifically adapting cage geometry and load distribution characteristics to the underlying endplate-specific density architecture. Intervertebral cages optimized to increase peripheral load transfer while minimizing stress concentration within central low-density regions may improve primary stability and reduce the risk of subsidence, thereby potentially enhancing long-term surgical outcomes in dogs affected by DA-CSM.

## Figures and Tables

**Table 1:** CT-OAM values with their corresponding SD for C6, its partitions, subdivisions, as well as their aggregate groups.

**C6**									
938.5									
117.5	**AF**								
	977.6								
	105.9	**AF-Dorsal-Sum**			**AF-Ventral-Sum**			**AF-**	**AF-**
		991.8			951.3			**Lateral-**	**Lateral-**
		127.7			115.8			**Left**	**Right**
		**AF-Dorsal-Left**	**AF-Dorsal-Center**	**AF-Dorsal-Right**	**AF-Ventral-Left**	**AF-Ventral-Center**	**AF-Ventral-Right**	991.4	988.5
		993.9	982.1	1003.3	923.4	980.1	946.4	110.7	121.7
		137.9	139.4	115.3	128.7	112.7	132.6		
	**NP**								
	900.5								
	139.4	**NP-Centrodorsal-Sum**		**NP-Ventrolateral-Sum**					
		852.5		933.3					
		140.9		140.3					
		**NP-Dorsal**	**NP-Central**	**NP-Left**	**NP-Ventral**	**NP-Right**			
		858.5	838.3	923.6	942.8	925.6			
		145.5	131.9	168.5	127	142			

**Table 2:** CT-OAM values with their corresponding SD for C7, its partitions, subdivisions, as well as their aggregate groups.

**C7**									
923									
116	**AF**								
	952.6								
	118.7	**AF-Dorsal-Sum**			**AF-Ventral-Sum**			**AF-**	**AF-**
		914.1			958.6			**Lateral-**	**Lateral-**
		109.7			126.9			**Left**	**Right**
		**AF-Dorsal-Left**	**AF-Dorsal-Center**	**AF-Dorsal-Right**	**AF-Ventral-Left**	**AF-Ventral-Center**	**AF-Ventral-Right**	992.8	1007.5
		908.3	956.8	855.8	946.6	976	942.6	155	148.9
		123.2	120.3	89.9	120.3	126.9	139.1		
	**NP**								
	902.4								
	122	**NP-Centrodorsal-Sum**		**NP-Ventrolateral-Sum**					
		836.5		948.5					
		128		123.7					
		**NP-Dorsal**	**NP-Central**	**NP-Left**	**NP-Ventral**	**NP-Right**			
		847	812	948.3	966.9	920.6			
		137.4	110.3	158	107.2	147.9			

**Table 3:** Comparison between categorized sum-groups of the affected cohort with the control cohort; significant difference marked by star.

Vertebral Level	Region	Group	Low Density *n* (%)	Intermediate Density *n* (%)	High Density *n* (%)	χ^2^	*p*-Value
C6	AF dorsal sum-group	Affected	2 (25.0)	2 (25.0)	4 (50.0)	4.180	0.1237
		Control	0 (0.0)	6 (40.0)	9 (60.0)		
C6	AF left lateral	Affected	1 (12.5)	4 (50.0)	3 (37.5)	3.792	0.1501
		Control	0 (0.0)	4 (26.7)	11 (73.3)		
C6	AF right lateral	Affected	2 (25.0)	1 (12.5)	5 (62.5)	4.632	0.0987
		Control	0 (0.0)	5 (33.3)	10 (66.7)		
C6	AF ventral sum-group	Affected	3 (37.5)	2 (25.0)	3 (37.5)	1.264	0.5314
		Control	5 (33.3)	7 (46.7)	3 (20.0)		
C6	NP centrodorsal sum-group	Affected	6 (75.0)	0 (0.0)	2 (25.0)	6.469	0.0394 *
		Control	10 (66.7)	5 (33.3)	0 (0.0)		
C6	NP ventrolateral sum-group	Affected	4 (50.0)	2 (25.0)	2 (25.0)	0.758	0.6846
		Control	7 (46.7)	6 (40.0)	2 (13.3)		
C7	AF dorsal sum-group	Affected	3 (37.5)	3 (37.5)	2 (25.0)	1.806	0.4053
		Control	3 (20.0)	10 (66.7)	2 (13.3)		
C7	AF left lateral	Affected	2 (25.0)	2 (25.0)	4 (50.0)	4.191	0.1230
		Control	0 (0.0)	4 (26.7)	11 (73.3)		
C7	AF right lateral	Affected	2 (25.0)	1 (12.5)	5 (62.5)	4.142	0.1261
		Control	0 (0.0)	2 (13.3)	13 (86.7)		
C7	AF ventral sum-group	Affected	3 (37.5)	1 (12.5)	4 (50.0)	4.160	0.1250
		Control	1 (6.7)	6 (40.0)	8 (53.3)		
C7	NP centrodorsal sum-group	Affected	6 (75.0)	0 (0.0)	2 (25.0)	4.903	0.0862
		Control	13 (86.7)	2 (13.3)	0 (0.0)		
C7	NP ventrolateral sum-group	Affected	3 (37.5)	1 (12.5)	4 (50.0)	2.769	0.2505
		Control	4 (26.7)	7 (46.7)	4 (26.7)		

## Data Availability

The full dataset is available from the corresponding author upon reasonable request.
